# Step-by-step optimisation of the radiosynthesis of the brain HDAC6 radioligand [^18^F]FSW-100 for clinical applications

**DOI:** 10.1186/s41181-024-00277-9

**Published:** 2024-06-03

**Authors:** Tetsuro Tago, Jun Toyohara

**Affiliations:** Research Team for Neuroimaging, Tokyo Metropolitan Institute for Geriatrics and Gerontology, 35-2 Sakae-Cho, Itabashi-Ku, Tokyo, 173-0015 Japan

**Keywords:** PET, Fluorine-18, Automated radiosynthesis, Copper-mediated radiofluorination, Brain, HDAC6

## Abstract

**Background:**

Histone deacetylase 6 (HDAC6) is an emerging target for the treatment and diagnosis of proteinopathies. [^18^F]FSW-100 was recently developed as a promising brain-penetrating radioligand for HDAC6 PET imaging and the process validation of [^18^F]FSW-100 radiosynthesis for clinical use is complete, but no detailed synthetic strategy nor process optimisation has been reported. Here, we describe the optimisation of several processes in [^18^F]FSW-100 radiosynthesis, including the ^18^F-fluorination reaction, semipurification of the ^18^F-intermediate, and purification of the product by high-performance liquid chromatography (HPLC), to achieve a radiochemical yield (RCY) adequate for clinical applications of the radioligand. Our findings will aid optimisation of radiosynthesis processes in general.

**Results:**

In the ^18^F-fluorination reaction, the amount of copper reagent was reduced without reducing the nonisolated RCY of the intermediate (50%), thus reducing the risk of copper contamination in the product injection solution. Optimising the solid-phase extraction (SPE) conditions for semipurification of the intermediate improved its recovery efficiency. The addition of anti-radiolysis reagents to the mobile phase for the HPLC purification of [^18^F]FSW-100 increased its activity yield in radiosynthesis using a high [^18^F]fluoride radioactivity of approximately 50 GBq. The SPE-based formulation method and additives for the injection solution were optimised, and the resulting [^18^F]FSW-100 injection solution was stable for over 2 h with a radiochemical purity of greater than 95%.

**Conclusions:**

Of all the reconsidered processes, we found that optimisation of the SPE-based semipurification of the intermediate and of the mobile phase for HPLC purification in particular improved the RCY of [^18^F]FSW-100, doubling it compared to that of the original protocol. The radioactivity of [^18^F]FSW-100 synthesized using the optimized protocol was sufficient for multiple doses for a clinical study.

**Supplementary Information:**

The online version contains supplementary material available at 10.1186/s41181-024-00277-9.

## Background

Histone deacetylase 6 (HDAC6) is recognised as a unique isoform in the HDAC family of proteins due to its location and functions. HDAC6 primarily localises in the cytoplasm, and deacetylates non-histone proteins such as α-tubulin, heat shock protein 90 and cortactin (Li et al. 2013). HDAC6 also binds polyubiquitinated proteins and transports them for degradation through the autophagy pathway (Kawaguchi et al. 2003; Rodriguez-Gonzalez et al. 2008). Pathological studies have shown an increase in HDAC6 expression in the brain of patients with various neurodegenerative proteinopathies such as Alzheimer’s disease (Ding et al. 2008; Odagiri et al. 2013), and selective inhibition of the enzyme is a potential therapeutic approach for such diseases (Guo et al. 2017; Onishi et al. 2021). In vivo imaging of HDAC6 by positron emission tomography (PET) can clarify spatiotemporal changes in its brain expression levels associated with the diseases. Thus, understanding these changes would facilitate the development of HDAC6-targeted drugs.

[^18^F]FSW-100 is a potential HDAC6 radioligand derived from the HDAC6 inhibitor SW-100 developed by Kozikowski et al. (2019) (Tago et al. 2021). It demonstrates favourable characteristics for HDAC6 imaging such as brain penetration in mice and high HDAC6 binding affinity (Tago et al. 2021). Additionally, it has been evaluated in non-human primates and shows good specific uptake in the brain (Tago et al. 2024). This previous paper also reported the process validation of an automated radiosynthesis strategy through three consecutive runs. The resulting injection solutions complied with the quality control criteria for clinical research. However, although the radiosynthesis procedure has been optimised since the first report (Tago et al. 2021), the details of the optimisation have not been described.

The radiosynthesis of [^18^F]FSW-100 uses copper-mediated radiofluorination (CMRF) and an arylboronic precursor. CMRF is an emerging method to radiolabel a variety of compounds through aromatic C-^18^F bonds. Radiosyntheses adopting this method have recently been reported for multiple clinically used PET probes such as [^18^F]FDOPA (Haider et al. 2022; Mossine et al. 2020; Nakaoka et al. 2022). Although the use of CMRF is expanding, it nonetheless faces several limitations, including vulnerability of the reaction to basic conditions and low radiochemical yield (RCY) in automated radiosynthesis (Mossine et al. 2015). Efforts to address these limitations, such as studies involving [^18^F]fluoride processing and ligands for copper reagents (Bowden et al. 2021; Hoffmann et al. 2023; Mossine et al. 2017) have improved CMRF, yet optimal conditions (e.g., reaction solvent and precursor/copper molar ratio) require further investigation for each substrate to obtain a good RCY.

**Fig. 1:**
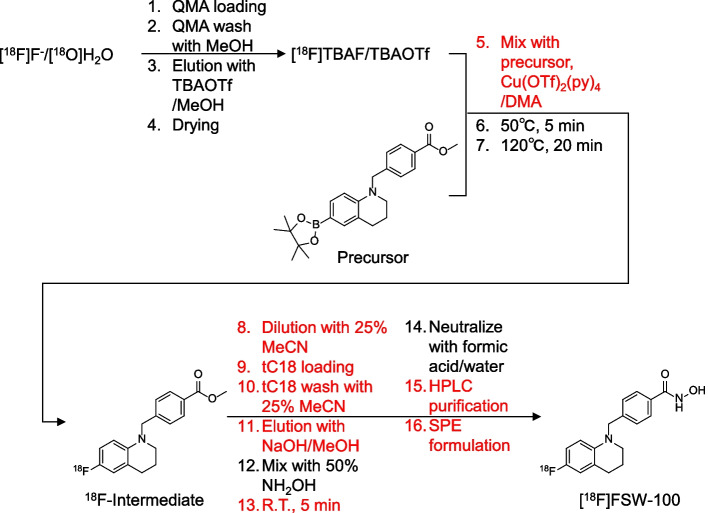
Schematic workflow of the radiosynthesis of [^18^F]FSW-100. The processes optimised in this study are coloured red

Here, we report details of the optimisation of an automated [^18^F]FSW-100 radiosynthesis not previously reported in our latest paper on process validation (Tago et al. 2024). Initially, the RCY of [^18^F]FSW-100 was only 4.5% following automated radiosynthesis and its activity yield was less than 1500 MBq despite using approximately 50 GBq of [^18^F]fluoride (Tago et al. 2021). An administration dose for humans is approximately 185 MBq, and thus this activity yield is insufficient to allow multiple PET scans. [^18^F]FSW-100 is radiosynthesised by a two-step reaction involving CMRF, followed by the formation of hydroxamic acid. We therefore decided to study processes other than CMRF to achieve a good RCY. In the present study, several radiosynthesis processes, including radiofluorination, semipurification of the intermediate, purification, and formulation, were investigated to identify key factors to improve the RCY (Fig. 1). Processes contributing to improved RCYs were indeed identified, indicating that the present study is informative as an approach to optimise automated radiosyntheses.

## Methods

### General

All reagents were commercially available and used without further purification unless otherwise indicated. The radiolabeling precursor and the reference standard of FSW-100 were prepared in-house (Tago et al. 2021) or by custom synthesis by BioDuro (San Diego, CA). Analytical high-performance liquid chromatography (HPLC) was performed using Prominence HPLC systems (Shimadzu, Kyoto, Japan) equipped with a radioactivity detector [US-3300 (Universal Giken, Odawara, Japan) or Gabi Star (Elysia-Raytest, Angleur, Belgium)].

### Copper-mediated radiofluorination reaction

[^18^F]Fluoride ions were produced by proton irradiation of ^18^O-enriched water (Taiyo Nippon Sanso, Tokyo, Japan) using an HM-20 cyclotron (Sumitomo Heavy Industries, Tokyo, Japan). ^18^F-Fluorination reaction studies were performed using a COSMiC-Mini radiosynthesiser (NMP Business Support, Sanda, Japan). An aqueous solution of [^18^F]fluoride ion (20–40 MBq) was passed through a Sep-Pak QMA Carbonate Light cartridge [46 mg sorbent, washed before use with 1 M KHCO_3_ (5 ml) and then water (8 ml); Waters, Milford, MA], then the cartridge was washed with anhydrous methanol (2 ml). The trapped [^18^F]fluoride ions were eluted with a methanol solution of tetrabutylammonium trifluoromethanesulfonate (TBAOTf) (19.2, 25.5, or 31.9 μmol/500 μl) into a reaction vial, then the solvent was removed by heating at 100 °C under a helium stream and reduced pressure. An *N*,*N*-dimethylacetamide (DMA) solution (500 μl) containing precursor (7.4 or 11 μmol) and Cu(OTf)_2_(py)_4_ (5.6 or 8.8 μmol) was added to the residue by reducing the pressure and allowing air to enter the reaction vial; this mixture was heated at 50 °C for 5 min and then at 120 °C for 20 min. The reaction mixture was cooled and mixed with a 25% acetonitrile aqueous solution (5 ml). The crude mixture was analysed by thin-layer chromatography (TLC) (TLC Silica gel 60 F254; Merck Millipore, Burlington, MA) with hexane/ethyl acetate (1/1) as the mobile phase. The plate was dried and exposed to a BAS-III imaging plate (Fujifilm, Tokyo, Japan) and an autoradiogram was obtained using an Amersham Typhoon Scanner IP (Global Life Sciences Technologies Japan, Tokyo, Japan). Data were analysed using ImageQuant TL (GE Healthcare, Buckinghamshire, UK).

### Semipurification of the ^18^F-intermediate

The optimisation study for the solid-phase extraction (SPE) semipurification of the ^18^F-intermediate involved the ^18^F-fluorination reaction using the method described in the previous section with a DMA solution (500 μl) containing precursor (7.4 μmol) and Cu(OTf)_2_(py)_4_ (5.6 μmol). The reaction mixture was cooled and mixed with water or a 25% acetonitrile aqueous solution (5 ml). The mixture was passed through a Sep-Pak tC18 Light cartridge [145 mg sorbent, washed before use with ethanol (5 ml) and then water (10 ml); Waters]. Water or a 25% acetonitrile aqueous solution (5 ml) was added to the reaction vial and then the solution was passed again through the cartridge. The trapped ^18^F-intermediate was eluted into another vial with methanol (500 μl). After measuring the radioactivity of the eluate and the SPE waste solution, the solutions were analysed by TLC-autoradiography (ARG) using the same method described above.

### Hydroxamic acid conversion

We studied the reaction conditions for converting the methyl ester moiety to hydroxamic acid by performing the ^18^F-fluorination reaction and ^18^F-intermediate trapping on a tC18 Light cartridge as described in the above section. The trapped ^18^F-intermediate was eluted with 0.6 M NaOH in methanol (1000 µl) or 1.2 M NaOH in methanol (500 µl) into a vial containing a 50% hydroxylamine aqueous solution (100 μl). After reacting for 5 min at room temperature, the reaction was quenched by adding formic acid (90 µl) and water (500 µl) and the mixture was analysed by HPLC [Column: Sunniest C18 (ChromaNik Technologies, Osaka, Japan), 5 μm, 4.6 × 150 mm; Eluent: acetonitrile/water/formic acid = from 45/55/0.1 (0–5 min) to 85/15/0.1 (8 min–); Flow rate: 1.0 ml/min; UV: 254 nm]. Conversion rates were calculated based on the peak areas of radioactivity chromatograms. The experiments were performed in duplicate.

### Mobile phase for semipreparative HPLC

This study was carried out using a CFN-MPS200 synthesiser (Sumitomo Heavy Industries). [^18^F]Fluoride ions were produced by proton irradiation of ^18^O-enriched water at 50 μA for 10 or 20 min using an HM-20 cyclotron, and the amount of radioactivity trapped on a QMA Carbonate Light cartridge was 26.4 ± 0.9 (n = 3) or 50.8 ± 2.0 (n = 5) GBq following 10 or 20 min of irradiation, respectively. The ^18^F-fluorination reaction and ^18^F-intermediate trapping on a tC18 Light cartridge were performed as described in the above section. The radioactivity of the cartridge was recorded using a radiodetector attachment on the synthesiser. The ^18^F-intermediate was eluted with 1.2 M NaOH in methanol (500 μl) into a second reaction vial containing a 50% hydroxylamine aqueous solution (100 μl). After 5 min at room temperature, formic acid (90 μl) and water (500 μl) were added and the mixture was purified by semipreparative HPLC on a system equipped with a radioactivity detector [Column: Sunniest C18 (ChromaNik Technologies), 5 μm, 10 × 250 mm; Eluent: ethanol/acetonitrile/water/formic acid = 5/35/60/0.1 or ethanol/acetonitrile/water/formic acid/250 mg/ml ascorbic acid solution = 5/35/60/0.1/1; Flow rate: 4.0 ml/min; UV: 254 nm for mobile phase without ascorbic acid or 320 nm for mobile phase with ascorbic acid] (Fig. S1). The fraction containing the product was mixed with a 10% ethanol aqueous solution (40 ml) and passed through an Oasis HLB Light cartridge [30 mg sorbent, washed before use with ethanol (5 ml) and then water (10 ml); Waters]. After washing the cartridge with a 10% ethanol aqueous solution (10 ml), the product was eluted with 1.4 ml of ethanol into a formulation vial preloaded with saline (12 ml), a 250 mg/ml ascorbic acid solution (550 μl; Sawai Pharmaceutical, Osaka, Japan), and polysorbate 80 (PS-80) (67 μl; MP Biomedicals, Irvine, CA). The solution was passed through a 0.22-μm sterilising filter (Millex GV; Merck Millipore), and its radioactivity was measured.

### SPE formulation of the HPLC-purified product

The preparation of a [^18^F]FSW-100 formulation by SPE involved comparing the utility of a Sep-Pak tC18 Light cartridge [washed before use with ethanol (5 ml) and then water (10 ml); n = 1] and an Oasis HLB Light cartridge [washed before use with ethanol (5 ml) and then water (10 ml); n = 3]. A semipreparative HPLC fraction containing [^18^F]FSW-100, prepared as described above, was diluted with 10% ethanol aqueous solution (> 40 ml) containing ascorbic acid (< 0.5% w/v). A portion of this solution was passed through an SPE cartridge and then washed with 10% ethanol aqueous solution (10 ml). The product was eluted with 1.4 ml of ethanol and the radioactivity of the eluate, cartridge, and waste solution was measured.

### The effect of formulation compositions on the stability of [^18^F]FSW-100

An ethanol solution (1.4 ml) of [^18^F]FSW-100 obtained by HPLC purification and SPE formulation using an HLB Light cartridge was prepared as described above. The ethanol solution was diluted with a formulation buffer containing additives, including ascorbic acid and PS-80. The radiochemical stability of the [^18^F]FSW-100 solution 2 h after completion of the synthesis was determined by analytical HPLC [Column: Sunniest C18, 5 μm, 4.6 × 150 mm; Eluent: acetonitrile/water/formic acid = 45/55/0.1 or 50/50/0.1; Flow rate: 1.0 ml/min; UV: 254 nm].

### Statistical analysis

Differences in the non-isolated RCY of the ^18^F-intermediate were analysed by one-way ANOVA with Tukey multiple comparison tests using GraphPad Prism Ver. 7.0 software (San Diego, CA). The recovery of [^18^F]FSW-100 radioactivity following HPLC purification was analysed by an unpaired *t* test using GraphPad Prism software.

## Results

### Optimisation of the copper-mediated radiofluorination reaction

First, we studied the optimal amounts of reagents for the CMRF of [^18^F]FSW-100 (Table 1) to improve the non-isolated RCY and to reduce the risk of contamination of injection solutions with impurities, such as copper and precursor-derived FSW-100 analogues. [^18^F]Fluoride was eluted from a QMA cartridge with a TBAOTf methanol solution (25.5 µmol/500 µl) in the previous report (Tago et al. 2021). After removing the solvent, a DMA solution (500 µl) containing 7.4 μmol precursor and 8.8 μmol Cu(OTf)_2_(py)_4_ was added to the residue, and the mixture was heated to 50 °C for 5 min and then at 120 °C for 20 min. This stepwise heating is adopted with reference to a paper by Mossine et al. to suppress competing reactions such as protodeboronation (Mossine et al. 2019). This reaction condition gave the ^18^F-intermediate with a non-isolated RCY of 49.0 ± 5.8% (entry 1). Changing the amount of TBAOTf did not affect the non-isolated RCY, while reducing it slightly decreased the elution efficiency of [^18^F]fluoride (entries 2 and 3). Reducing the amount of copper reagent from 8.8 to 5.6 µmol did not affect the non-isolated RCY (entry 4). Using 5.6 µmol Cu(OTf)_2_(py)_4_, increasing the precursor amount by 50% resulted in a slight increase in the non-isolated RCY (entry 5). Overall, no statistically significant differences were observed in the non-isolated RCY using the tested conditions and thus we chose entry 4 as the optimal condition because it required lower amounts of reagent.

**Table 1:** Optimisation of reagent amounts for CMRF

Entry	TBAOTf (µmol)	^18^F-elution efficiency (%)	Precursor (μmol)	Cu(OTf)_2_(py)_4_ (μmol)	Non-isolated RCY of ^18^F-intermediate (%)	*n*
1	26	96 ± 2	7.4	8.8	49.0 ± 5.8	5
2	19	90, 96	7.4	8.8	54.4, 56.4	2
3	32	94, 95	7.4	8.8	51.3, 56.1	2
4	26	96 ± 1	7.4	5.6	50.4 ± 3.8	3
5	26	96 ± 1	11	5.6	57.4 ± 3.5	3

Data are expressed as mean ± standard deviation (SD)

### Optimisation of SPE semipurification of the ^18^F-intermediate

**Fig. 2:**
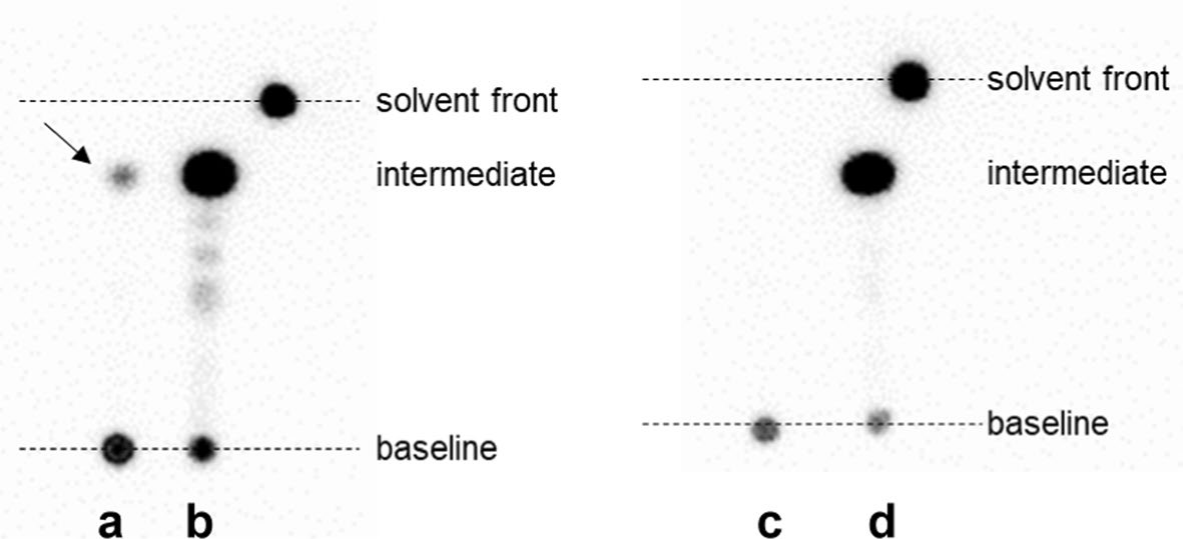
TLC-ARG analysis for optimising SPE semipurification of the ^18^F-intermediate. **a** and **b** TLC-ARG images of an SPE waste solution (**a**) and methanol eluent (**b**) when the reaction mixture was diluted with water. The ^18^F-intermediate was observed in the waste solution (arrow); **c** and **d** TLC-ARG images of an SPE waste solution (**c**) and methanol eluent (**d**) when the reaction mixture was diluted with 25% acetonitrile

We optimised the SPE process to remove the copper reagent after the ^18^F-fluorination reaction. In a previous report (Tago et al. 2021), a reaction mixture containing the ^18^F-intermediate was diluted with water and passed through a Sep-Pak C8 Short cartridge (Waters). After washing the cartridge with water, the trapped ^18^F-intermediate was eluted with 0.6 M NaOH in methanol into a second reaction vial. The present optimisation study revealed that a significant amount of the ^18^F-intermediate passed through the cartridge and leaked into the waste solution during the loading and/or cartridge washing steps (data not shown). We minimised the loss of the ^18^F-intermediate by using a tC18 Light cartridge instead of the C8 cartridge, as this cartridge should better retain the ^18^F-intermediate. In addition, we compared the effect of water containing 25% acetonitrile for diluting the reaction mixture and to wash the cartridge. TLC-ARG showed that the use of pure water resulted in the SPE waste solution containing some ^18^F-intermediate (Fig. 2) and its radioactivity was about 15% of the initial radioactivity of the [^18^F]fluoride ion (corrected for decay) (Table 2). In contrast, no ^18^F-intermediate was observed in the SPE waste solution when a 25% acetonitrile aqueous solution was used for elution. The residual radioactivity in the cartridge after elution of the ^18^F-intermediate was less than 2% relative to the initial radioactivity of the [^18^F]fluoride ion regardless of the eluent (Fig. S2). We thus decided that 25% acetonitrile aqueous solution is more suitable for this SPE semipurification step.

**Table 2:** Optimisation of SPE semipurification of the ^18^F-intermediate

Entry	Dilution of the reaction mixture	Cartridge wash	Loss of ^18^F-intermediate in SPE waste solution (%)^a^	n
1	H_2_O 5 ml	H_2_O 5 ml	15.3, 16.0	2
2	25% acetonitrile 5 ml	25% acetonitrile 5 ml	Negligible	2

^a^Proportion of the radioactivity of ^18^F-intermediate in the SPE waste solution relative to that of the initial [^18^F]fluoride ion (decay corrected)

### Conditions for hydroxamic acid conversion

**Fig. 3:**
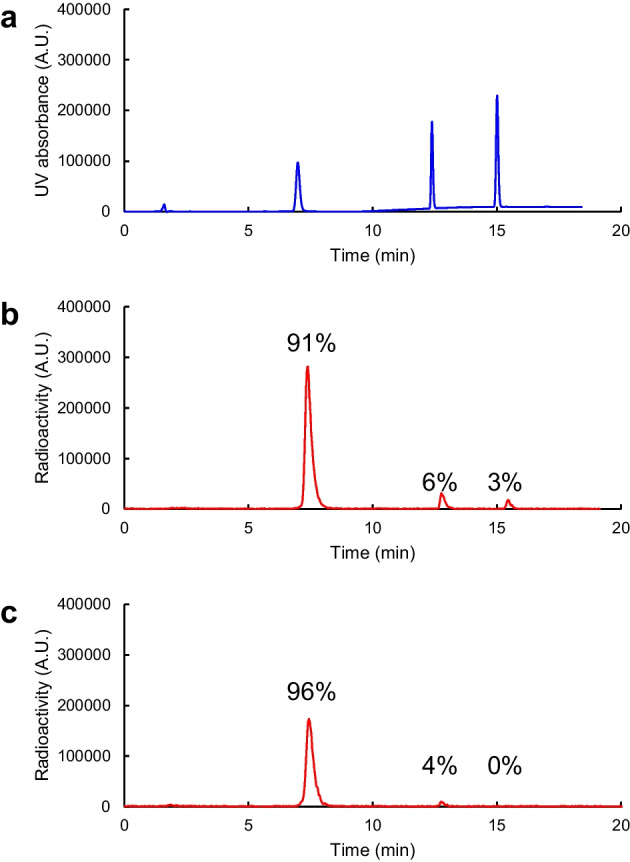
Analytical HPLC chromatograms of hydroxamic acid conversion. **a** UV chromatogram of a reference standard solution containing FSW-100 (7.0 min), the carboxylic acid form of FSW-100 (12 min), and the intermediate (15 min). **b** and **c** Radioactivity chromatograms of crude reaction solutions using 1000 µL of 0.6 M NaOH methanol solution (**b**) or 500 µL of 1.2 M NaOH methanol solution (**c**). Conversion efficiencies are expressed as the mean of two experiments

Changing the cartridge size (from a C8 Short cartridge containing 400 mg of sorbent to a tC18 Light cartridge containing 145 mg of sorbent) reduced the amount of solution required to elute the ^18^F-intermediate, so we studied the reaction conditions for the conversion of hydroxamic acid. We compared 0.6 M NaOH in 1000 µl methanol, as used in the previous work (Tago et al. 2021), and 1.2 M NaOH in 500 µl methanol as an eluent. Eluates containing the ^18^F-intermediate were reacted with hydroxylamine, and the efficiency of hydroxamic acid conversion was measured after 5 min reaction, a reduction from 10 min in the previous work. Using 1000 µl of 0.6 M NaOH, a small amount of the ^18^F-intermediate (3%) remained after the 5 min reaction and the conversion efficiency was 91% (Fig. 3), whereas the use of 500 µl of 1.2 M NaOH completely consumed the ^18^F-intermediate and the conversion efficiency was 96%, suggesting that an increase in reagent concentration helped improve conversion.

### Mobile phase optimization for semipreparative HPLC

Given that the RCY of [^18^F]FSW-100 can be reduced due to its radiolysis during HPLC purification, we investigated an optimal mobile phase for semipreparative HPLC (Table 3). We evaluated the effects of the radioactivity level of the starting [^18^F]fluoride ion on RCY by comparing low (26 GBq) and high (51 GBq) levels. When a high radioactivity level was applied, the amount of ^18^F-intermediate radioactivity trapped on the SPE cartridge after the ^18^F-fluorination reaction was double that obtained using a low radioactivity level (from 7.5 to 15 GBq), suggesting that the non-isolated RCY was unaltered by the radioactivity level. However, the activity yield of [^18^F]FSW-100 after HPLC purification using a high radioactivity level was only 1.4 times higher than that obtained using a low level. The percent radioactivity of HPLC-purified [^18^F]FSW-100 versus that of the ^18^F-intermediate trapped on an SPE cartridge (decay corrected) was 21% and 13% for the low and high levels of radioactivity, respectively. Since the conversion rate in hydroxamic acid formation is the same at both radioactivity levels, these results suggest the loss of [^18^F]FSW-100 due to radiolysis during the HPLC purification process. We increased the stability of [^18^F]FSW-100 during purification by adding ascorbic acid to the HPLC mobile phase as an anti-oxidant (Scott et al. 2009), which significantly improved the recovery of [^18^F]FSW-100 radioactivity when using the high radioactivity level. The recovery rate of HPLC-purified [^18^F]FSW-100 compared to the SPE-trapped ^18^F-intermediate radioactivity was 26%.

**Table 3:** Optimisation of the mobile phase for semipreparative HPLC

Entry	RA of [^18^F]fluoride ion (GBq)	RA retained on tC18 (GBq)	Semiprep. HPLC mobile phase	Rate of the product RA to the RA retained on tC18 (%)^a^	[^18^F]FSW-100 activity yield (GBq)	n
1	26.4 ± 0.9	7.5 ± 1.4	Without AsA	21.0 ± 0.7	1.1 ± 0.2	3
2	51.0, 51.7	16.3, 17.7	Without AsA	14.0, 11.8	1.7, 1.4	2
3	50.4 ± 2.7	13.6 ± 1.6	With AsA^b^	26.4 ± 3.8*	2.6 ± 0.6	3

^a^Proportion of the radioactivity of HPLC-purified [^18^F]FSW-100 relative to that trapped on a tC18 cartridge in SPE-semipurification of the ^18^F-intermediate (corrected for decay)

Data are expressed as mean ± SD

^b^0.25% (w/v)

^*^p < 0.05 versus entry 2

*RA* Radioactivity; *AsA* Ascorbic acid

### SPE formulation study of the HPLC-purified product

**Fig. 4:**
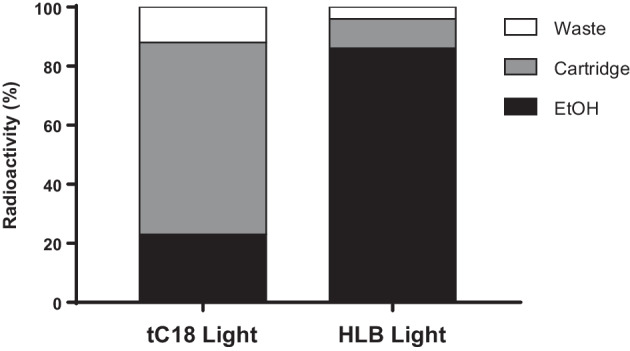
Distribution of radioactivity in the SPE formulation process. The radioactivity of the “Waste” solution, “Cartridge”, and “EtOH” eluent was measured after SPE and their proportions were calculated

We tested the SPE-based formulation of HPLC-purified [^18^F]FSW-100 in place of the evaporation drying method reported previously (Tago et al. 2021). The present study investigated two types of SPE cartridges: Sep-Pak tC18 Light and Oasis HLB Light (Waters) (Fig. 4). Diluting a semipreparative HPLC fraction containing [^18^F]FSW-100 with a 10% ethanol aqueous solution and passing it through a tC18 Light cartridge resulted in 88% of the total radioactivity of the HPLC fraction being trapped in the cartridge, but 65% of the total radioactivity remained in the cartridge after elution with 1.4 ml of ethanol. In contrast, using an HLB Light cartridge, 96 ± 1% of the total radioactivity was trapped and 86 ± 4% was recovered by ethanol elution, suggesting that this cartridge is more suitable for generating a [^18^F]FSW-100 formulation.

### Effects of formulation composition on product stability

**Fig. 5:**
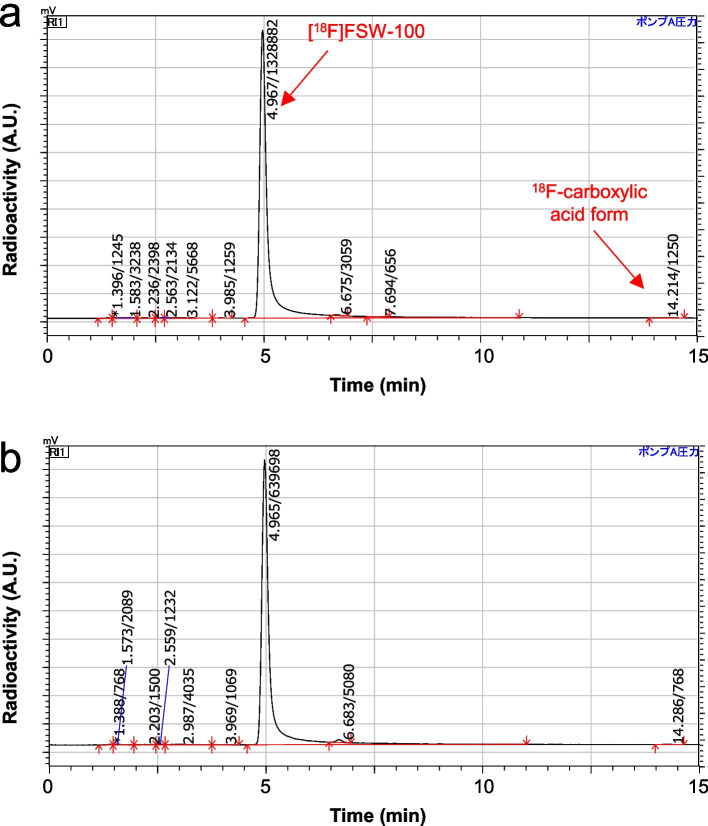
Representative analytical HPLC chromatograms for an [^18^F]FSW-100 injection solution. **a** and **b** Radioactivity chromatograms for the formulation shown as entry 3 in Table 4 at 0 h (**a**) and 2 h (**b**) after the end of synthesis

The effects of formulation composition on the stability of [^18^F]FSW-100 were evaluated (Table 4). An ethanol eluate containing [^18^F]FSW-100 obtained using the SPE-based formulation was diluted with saline containing ascorbic acid, resulting in approximately 8.5% v/v and 0.6% w/v of ethanol and ascorbic acid in the formulation, respectively. This formulation gave a radiochemical purity (RCP) of 96.8% at the end of the synthesis (entry 1) but the purity decreased to 54.7% 2 h after synthesis. The analytical HPLC radioactivity chromatogram 2 h after synthesis shows multiple peaks of highly polar impurities and a carboxylic acid form of [^18^F]FSW-100 (Figs. S3 and S4). We improved the stability of [^18^F]FSW-100 by increasing the proportion of additives. Setting the ethanol and ascorbic acid at approximately 10% v/v and 1.0% w/v, respectively, resulted in an RCP of around 95% even 2 h after the end of synthesis (entry 2). We also evaluated the stabilizing effects of adding PS-80 to the [^18^F]FSW-100 formulation. PS-80 is used in some radiopharmaceuticals to facilitate the solubilisation of lipophilic compounds and to prevent absorption of compounds into materials used in the synthesis, such as tubes and filters (Yip et al. 2015). Adding PS-80 at 0.1 or 0.5% v/v to the [^18^F]FSW-100 formulation slightly improved stability and maintained the RCPs around 97% over 2 h after the end of synthesis (entries 3 and 4) (Fig. 5). We therefore selected entry 3 in Table 4 as the preferred formulation for [^18^F]FSW-100.

**Table 4:** Optimisation of a formulation buffer for [^18^F]FSW-100 injection solution

Entry	EtOH (% v/v)^a^	AsA (% w/v)^a^	PS-80 (% v/v)^a^	RA conc. (MBq/ml)^b^	RCP (%)			pH	n
					At 0 h	At 1 h	At 2 h		
1	8.5	0.6	0	80, 88	96.3, 97.2	− , 56.9	75.3, 34.1	7.1^c^	2
2	10	1.0	0	173, 174	97.8, 98.0	− , 95.5	96.4, 95.1	6.7	2
3	10	1.0	0.1	240 ± 90	98.4 ± 0.3	97.3 ± 0.5	97.1 ± 0.5	6.7	3
4	10	1.0	0.5	146 ± 52	98.3 ± 0.5	96.6 ± 1.4	97.4 ± 0.6	6.6	6

^a^Theoretical values

^b^At the end of the synthesis

^c^n = 1

Data are expressed as mean ± SD

*AsA* Ascorbic acid; *RA conc.* Radioactivity concentration

## Discussion

CMRF is a powerful tool for introducing [^18^F]fluoride into aromatic ring-containing PET imaging probes, and many arylboronic precursors have been radiofluorinated since the first use of CMRF in 2014 (Tredwell et al. 2014; Wright et al. 2020). However, this type of reaction often provides unsatisfactory RCY following automated radiosynthesis compared to the nonisolated RCY observed following manual synthesis (Mossine et al. 2015, 2018). There are several possible reasons for this discrepancy, including loss of reagent in tubing, but they likely vary depending on the substrate and equipment used. In the present study, we improved the RCY of automated [^18^F]FSW-100 radiosynthesis utilizing the CMRF. Radiosynthesis consists of multiple processes, including hydroxamic acid formation and product purification, in addition to CMRF, and thus we optimised each of these processes.

Acceptable daily exposure for the parenteral administration of copper defined by the International Council for Harmonisation Guideline for Elemental Impurities (Q3D) (2022) is 340 μg/day or 34 μg/ml for an injection volume of 10 ml. We minimised the risk of the copper content exceeding this limit in the [^18^F]FSW-100 injection formulation by studying the effect of reducing the amount of Cu(OTf)_2_(py)_4_ in non-isolated RCYs of the ^18^F-intermediate. For this copper-reduced condition, we set the amount of Cu(OTf)_2_(py)_4_ at 5.6 μmol, corresponding to 356 μg of copper. In the present study, radiofluorination with less than 5.6 µmol of Cu(OTf)_2_(py)_4_ was not performed. In general, copper-mediated radiofluorination tends to decrease RCY as the equivalence ratio of Cu(OTf)_2_(py)_4_ to the precursor decreases (Bowden et al. 2021, 2024). If the volume of the final solution of [^18^F]FSW-100 is 12 mL or more, the copper concentration will not exceed the upper limit of 30 μg/mL, even if all of the copper used in the reaction were mixed into the injection solution. We found that reducing the copper reagent from 8.8 to 5.6 μmol did not affect the non-isolated RCY (Table 1). In three consecutive production validation runs reported in our recent paper (Tago et al. 2024), we confirmed that the amount of copper in an [^18^F]FSW-100 injection solution was below the detection limit (< 0.1 µg/mL) of inductively coupled plasma mass spectrometry.

The radiosynthesis of [^18^F]FSW-100 requires semipurification of the ^18^F-labelled intermediate between CMRF and hydroxamic acid formation. Previous work demonstrated that the hydroxamic acid formation reaction in the presence of copper reagent can hydrolyse the methyl ester group (Tago et al. 2021). Diluting the reaction solution with a 25% acetonitrile aqueous solution improved retention of the intermediate on a cartridge compared to when the reaction mixture was diluted with water (Table 2), likely due to the lipophilic ^18^F-intermediate precipitating with precursor-derived compounds upon dilution with water. Aqueous acetonitrile might increase the solubility of the ^18^F-intermediate, thus increasing the interaction of the compound with the resin in the cartridge.

The mobile phase for semipreparative HPLC was optimised to improve the stability and recovery of [^18^F]FSW-100 during purification. Hydroxamic acids can be oxidised by radicals to transient nitroxide radicals, which produce the respective carboxylic acids via oxidation and hydrolysis (Goldstein & Samuni 2015). Radiolysis during HPLC purification was reported for the adamantane-based HDAC6 radioligand [^18^F]EKZ-001, and contamination by its carboxylic acid form reduced the RCP of an HPLC-purified fraction (Celen et al. 2020). The authors improved the stability of [^18^F]EKZ-001 by replacing acetonitrile in the mobile phase with ethanol, which has radical scavenger properties. The mobile phase for the HPLC purification of [^18^F]FSW-100 contained 5% ethanol and 35% acetonitrile, as reported previously (Tago et al. 2021). However, it was difficult to replace acetonitrile with ethanol due to the increase in column pressure and thus ascorbic acid was added to the mobile phase as an anti-radiolysis reagent. This modification significantly improved the recovery rate of [^18^F]FSW-100 (Table 3).

We formulated [^18^F]FSW-100 using an SPE method instead of the evaporation method used in previous work (Tago et al. 2021) from a perspective of Good Manufacturing Practice (Lemaire et al. 1999). In the present study, tC18 Light and HLB Light cartridges were tested for the SPE step. Both cartridges retained [^18^F]FSW-100 well but the elution efficiency was better with an HLB cartridge than with a tC18 cartridge (Fig. 4). tC18 and HLB are reversed-phase cartridges containing silica-based resin and copolymer-based resin, respectively. Interactions between the surface silanol groups on the particles in a tC18 cartridge and [^18^F]FSW-100, which has a basic moiety, can make elution difficult. The difference in the amount of resin contained in the cartridges may also affect elution efficiency (tC18 Light: 145 mg; HLB Light: 30 mg).

The stability of the [^18^F]FSW-100 injection formulation was improved by optimising the amounts of additives. Increasing the proportions of ethanol and ascorbic acid, which have radical scavenging properties, improved the stability of [^18^F]FSW-100 (entry 2). Interestingly, adding PS-80 to the injection formulation also slightly improved stability (entries 3 and 4). Although the effect was small and the underlying mechanism was unclear, we nonetheless selected the composition shown as entry 3 as being optimal. PS-80 is a common excipient for medicinal products and its maximum intravenous dose as a pharmaceutical excipient is 500 mg in Japan (Japanese Pharmaceutical Excipients Directory. 2021). For example, PS-80 is used to solubilize hydrophobic drugs such as docetaxel (ten Tije et al. 2003). PS-80 is also used for the formulation of brain imaging PET drugs, often at concentrations of approximately 0.5% in injectable solutions (Maruyama et al. 2013; Nelissen et al. 2009; Toyohara et al. 2020). It remains unknown whether PS-80 functions as a radical scavenger (Perez-Roses et al. 2015).

One of the challenges in the present study was optimising SPE conditions. Although SPE is useful for purification and formulation processes in automated synthesis of radioligands, finding conditions suitable for retention and elution of compounds that have both hydrophilic and lipophilic functional groups, such as HDAC6 radioligands, often requires consideration of various conditions. Additionally, optimising the formulation composition to provide high radiochemical stability was also a challenge with [^18^F]FSW-100. Maintaining radiochemical stability might be a common issue for hydroxamic acid-based radioligands, as [^18^F]EKZ-001 also required optimisation of the HPLC mobile phase and reformulation, as mentioned above (Celen et al. 2020). We hope that the information on SPE and formulation optimisation described in the present study will help optimise and automate the radiosynthesis of other HDAC imaging ligands, such as [^18^F]PB118, another HDAC6 radioligand synthesised using CMRF (Bai et al. 2022).

The results of three consecutive production validation runs of [^18^F]FSW-100 radiosynthesis using the optimised conditions (Table S1) were previously reported elsewhere (Tago et al. 2024). The time required for the synthesis was reduced from 107 ± 3 min (n = 6) using the previous protocol (Tago et al. 2021) to 94 ± 1 min (n = 3) using the proposed protocol. RCY was 11.5 ± 1.6% and the activity yield was 3303 ± 413 MBq from a starting [^18^F]fluoride activity of about 50 GBq. The obtained radioactivity is adequate for clinical research purposes using an administration dose of 185 MBq. Note that the radiosynthesis optimisations described here used a CFN-MPS200 radiosynthesiser; further modifications may be required for radiosynthesis using a different synthesiser to obtain a good RCY.

## Conclusions

In summary, we optimised the radiosynthesis of [^18^F]FSW-100 to achieve sufficient RCY for clinical application. The proposed processes involved optimising the SPE-based semipurification of the ^18^F-intermediate and optimising the mobile phase for HPLC-purification of [^18^F]FSW-100, both of which particularly improved the RCY. Furthermore, we determined a formulation that stabilises [^18^F]FSW-100 for injection. The resulting RCY obtained by radiosynthesis employing the described optimised processes was double that of the original protocol and was adequate for providing multiple doses of [^18^F]FSW-100 for clinical studies.

### Supplementary Information

Supplementary Material 1.

## Data Availability

The datasets used and/or analysed during the current study are available from the corresponding author on reasonable request.

## Abbreviations

ARG: Autoradiography
CMRF: Copper-mediated radiofluorination
DMA: *N*, *N*-Dimethylacetamide
HDAC: Histone deacetylase
HPLC: High-performance liquid chromatography
PET: Positron emission tomography
PS-80: Polysorbate 80
RCP: Radiochemical purity
RCY: Radiochemical yield
SPE: Solid-phase extraction
TBAOTf: Tetrabutylammonium trifluoromethanesulfonate
TLC: Thin-layer chromatography
