# Supplementary material for: Step-by-step optimisation of the radiosynthesis of the brain HDAC6 radioligand [18F]FSW-100 for clinical applications
Source: EJNMMI Radiopharm Chem. 2024 Jun 3;9:45. doi: 10.1186/s41181-024-00277-9 (PMC11147973; doi:10.1186/s41181-024-00277-9)
Supplement: Supplementary file 1 — Supplementary Material 1. [file 41181_2024_277_MOESM1_ESM.pdf]

Supporting Information

Step-by-step optimisation of the radiosynthesis of the brain HDAC6 radioligand [^{18}F]FSW-100 for clinical applications

Tetsuro Tago, Jun Toyohara*

Research Team for Neuroimaging, Tokyo Metropolitan Institute for Geriatrics and Gerontology, 35-2 Sakae-cho, Itabashi-ku, Tokyo 173-0015, Japan

*Corresponding author

toyohara@pet.tmig.or.jp

Table S1 Optimized automated radiosynthesis of [^{18}F]FSW-100 using a CFN-MPS200 synthesiser

Procedure
1. Pass ^{18}O-enriched water containing [^{18}F]fluoride through a QMA Carbonate Light cartridge. Wash the cartridge with 2 ml of methanol.
2. Elute [^{18}F]fluoride into a reaction vial with a TBAOTf methanol solution (25.5 µmol/500 µl). Remove the solvent by heating at 100 °C under a nitrogen stream and reduced pressure.
3. Add a mixture of precursor (7.4 µmol), $Cu(OTf)_2(py)_4$ (5.6 µmol), and DMA (500 µl) to the residue and allow air to enter the reaction vial.
4. Heat the vial stepwise to 50 °C for 5 min and then at 120 °C for 20 min.
5. Mix 5 mL of 25% acetonitrile aqueous solution with the reaction mixture and pass the mixture through a tC18 Light cartridge. Add 5 mL of 25% acetonitrile aqueous solution to the vial and pass the solution through the cartridge again.
6. Elute the trapped ^{18}F-intermediate with 500 µl of 1.2 M NaOH methanol solution into another reaction vial containing 100 µl of 50% hydroxylamine aqueous solution. After 5 min at room temperature, add 90 µl of formic acid and 500 µl of water.

7. Purify the mixture by semipreparative HPLC [Column: Sunniest C18, 5 µm, 10 × 250 mm (ChromaNik Technologies); Eluent: ethanol/acetonitrile/water/formic acid/250 mg/ml ascorbic acid solution = 5/35/60/0.1/1; Flow rate: 4.5 ml/min; UV: 320 nm].
8. Dilute a fraction containing the product with 40 ml of 10% ethanol aqueous solution and pass the mixture through an HLB Light cartridge. Wash the cartridge with 10 ml of 10% ethanol aqueous solution.
9. Elute the product with 1.4 ml of ethanol and mix it with saline (12 ml), a 250 mg/ml ascorbic acid solution (550 µl), and polysorbate-80 (13 µl).
10. Transfer the mixture via a 0.22-µm sterilising filter to a sterile empty vial.

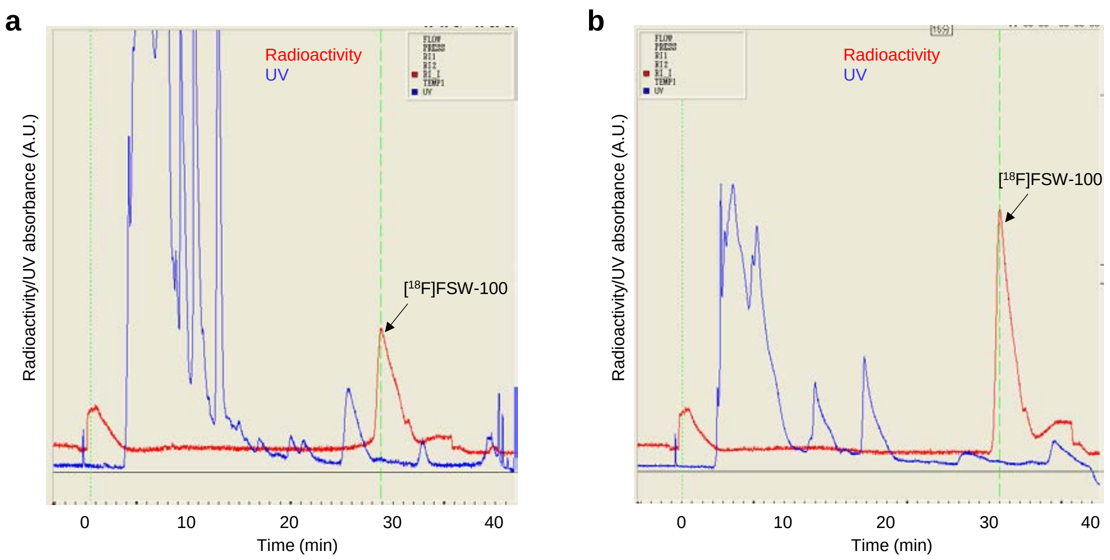

Fig. S1 Preparative HPLC chromatograms for the purification of [^{18}F]FSW-100. **a** Mobile phase without ascorbic acid (entry 2 in Table 3); **b** Mobile phase with ascorbic acid (entry 3 in Table 3). UV absorbance wavelengths are 254 and 320 nm for **a** and **b**, respectively

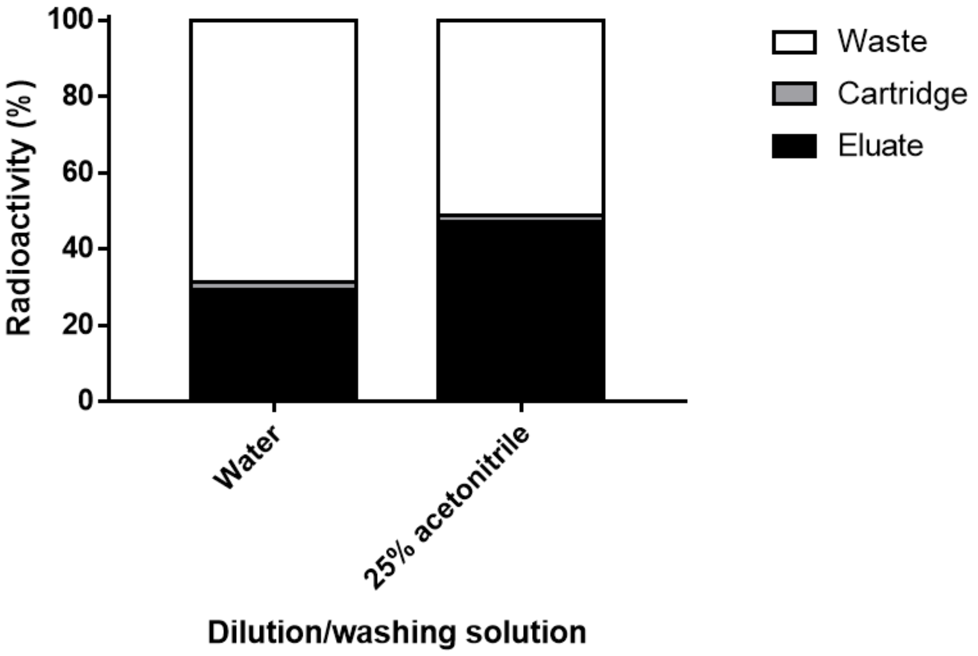

Fig. S2 Distribution of radioactivity in the semipurification process of the ^{18}F-intermediate. Radioactivity of the "Waste" solution, "Cartridge", and methanol "Eluate" was measured after SPE, and their proportions were calculated

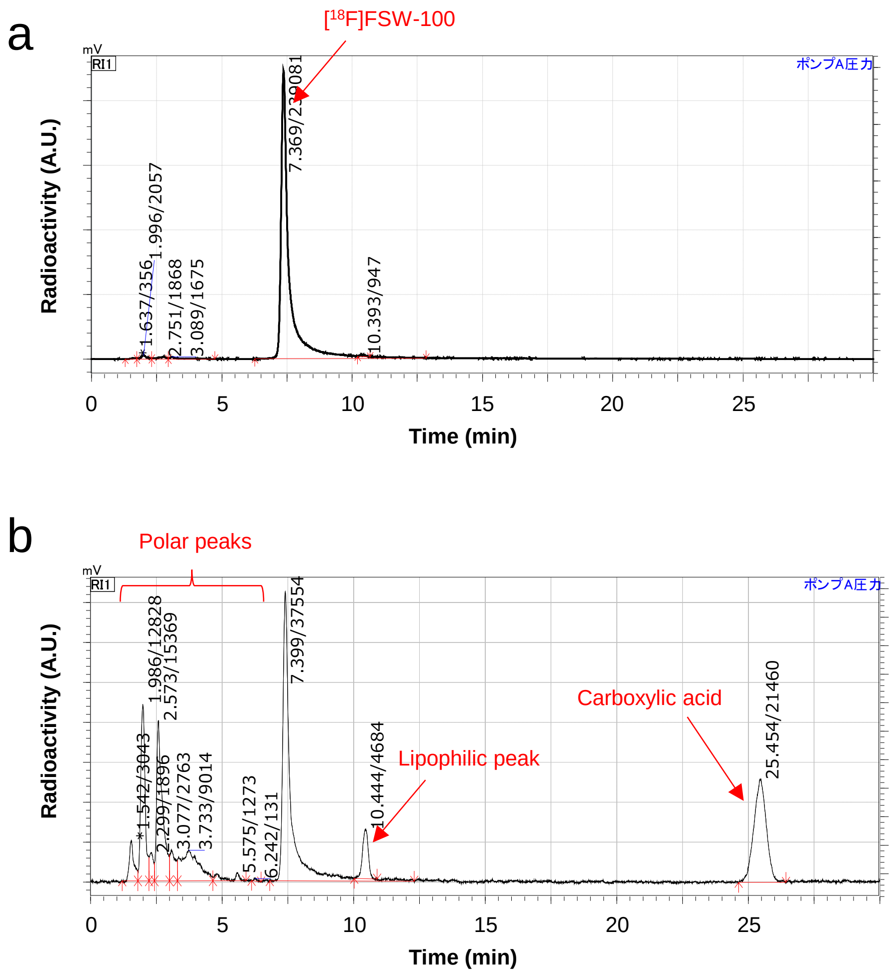

Fig. S3 Representative analytical HPLC chromatograms of the [^{18}F]FSW-100 injection formulation shown as entry 1 in Table 4. Radioactivity chromatograms at 0 h (**a**) and 2 h (**b**) after the end of synthesis are shown. Note that the mobile phase for these chromatograms (acetonitrile/water/formic acid = 45/55/0.1) is different from that used in Fig. 4 (acetonitrile/water/formic acid = 50/50/0.1), and thus the retention time of [^{18}F]FSW-100 is different for the two figures

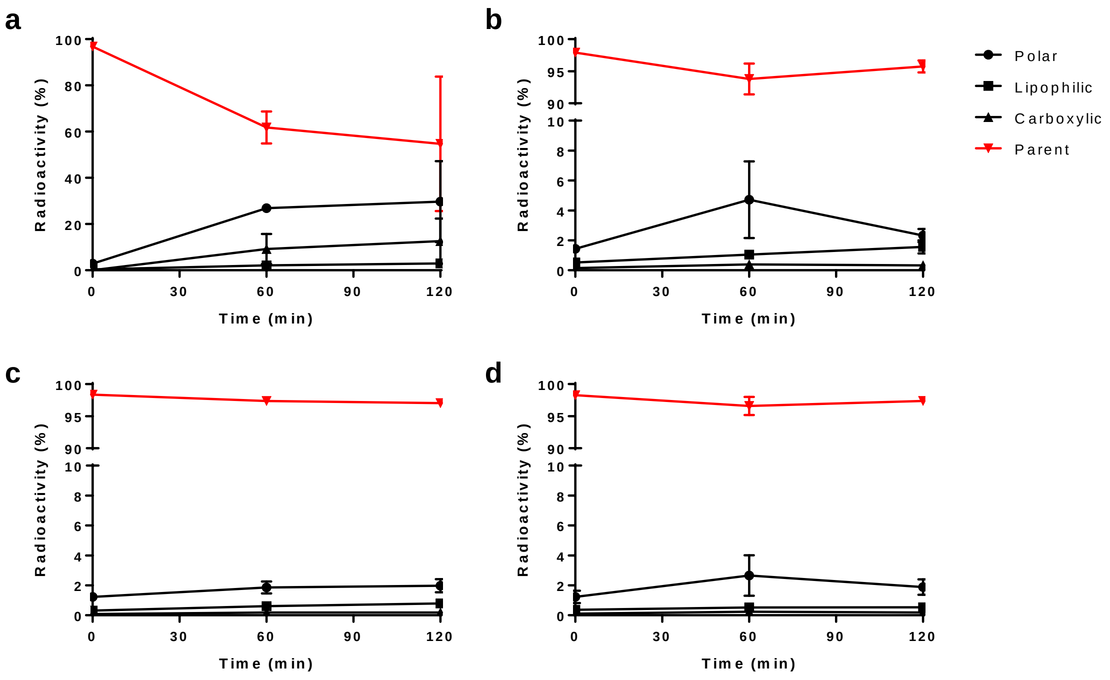

Fig. S4 The stability of the [^{18}F]FSW-100 injection formulations shown in Table 4 (**a** entry 1; **b** entry 2; **c** entry 3; **d** entry 4). Time-radioactivity curves of the parent [^{18}F]FSW-100 and of radioactive impurities up to 120 min after the end of synthesis are shown. The radioactive impurities (polar, lipophilic, and carboxylic) correspond to the peaks in the radio-HPLC chromatogram shown in Fig. S2b
